# Localization of a single tactile stimulus during saccadic eye movements

**DOI:** 10.1177/20416695251376196

**Published:** 2025-09-12

**Authors:** Kazumichi Matsumiya, Nanami Nakashima

**Affiliations:** 1 Graduate School of Information Sciences, 13101 Tohoku University, Japan

**Keywords:** touch, eye movements, saccade, spatial perception

## Abstract

To localize tactile events in external space, our perceptual system must transform skin-based locations into an external frame of reference. Such a transformation has been reported to involve reference frames that are unrelated to tactile sensations, such as eye position, which supports the idea that a visual reference frame is a single unified frame of reference for transforming spatial information from all sensory modalities. However, it remains unclear how tactile events are perceptually localized during saccadic eye movements. In this study, we presented a single tactile stimulus at a fixed location on the skin and investigated the time course of its localization before, during, and after a saccade. Participants reported the perceived location of the tactile stimulus in a visually aligned virtual space. We found that the tactile stimulus was mislocalized in the direction of the saccade. This mislocalization appeared even before the presentation of the saccade target and continued until 500 ms after saccade onset. These findings demonstrate that tactile localization is influenced by saccade planning or preparation and suggest that the time course of tactile localization during a saccade may differ from previously reported patterns of visual localization during a saccade.

## How to cite this article

Kazumichi Matsumiya, Nanami Nakashima. (2025). Localization of a single tactile stimulus during saccadic eye movements. *i-Perception*, 16(5), 1–25. https://doi.org/10.1177/20416695251376196

## Introduction

Humans perceive tactile events in the external world through active exploration (Gibson 1962). Tactile events often occur on the skin while body parts (e.g., limbs) move in space. The spatial locations of tactile events on the limb are initially elicited by sensory receptors on the skin in a skin-based reference frame. However, when the limb moves in space, the perceptual system must integrate the skin location with the current limb posture to localize the tactile events in external space. Thus, tactile localization requires the transformation of the initial skin-based location into an external reference frame that accounts for body posture (Heed et al. 2015).

Nevertheless, tactile localization has been reported to involve reference frames that are not directly related to tactile sensations, such as eye position (Harrar and Harris 2009). Harrar and Harris (2009) showed that the perceived location of a touch was shifted by a change in eye position, which suggests that the perceptual system integrates skin location with current eye position for tactile localization. This finding may be explained by the standard theory of multisensory integration, which posits that the perceptual system converts spatial information from all sensory modalities into a retinal reference frame (Pouget, Deneve, et al. 2002; Pouget, Ducom, et al. 2002). Pouget, Ducom, et al. (2002) found that the location of a reaching target is encoded in retinal coordinates, regardless of whether the targets are visual, auditory, or proprioceptive. Their findings are surprising because the location of a reaching target can be computed from a shoulder-based reference frame without recovering the retinal location of the target. If the retinal reference frame is a common frame across all sensory modalities, the perceptual system would require knowledge of eye position to convert tactile information into the common retinal reference frame, which is supported by the results of Harrar and Harris (2009).

While the standard theory of multisensory integration posits that tactile spatial information relies on a common retinal reference frame, recent research suggests that tactile localization during saccades may instead depend on alternative spatial representations. Overvliet et al. (2011) reported that saccadic responses to tactile stimuli were slower when participants’ hands were crossed compared to when they were uncrossed (Overvliet et al. 2011). This finding indicates that body posture modulates the interaction between saccades and tactile spatial processing, implying that tactile localization may rely on a modality-specific rather than a common retinal reference frame. This challenges the standard theory of multisensory integration, which assumes a common retinal reference frame across all sensory modalities, and highlights the need to investigate how saccades influence tactile localization. Therefore, saccades offer a valuable opportunity to test whether tactile localization depends on a common or modality-specific retinal reference frame.

cTo our knowledge, however, no study has attempted to address how tactile stimuli are perceptually localized before, during, and after the execution of saccadic eye movements. Previous studies have investigated saccades guided by tactile or proprioceptive stimuli (Amlot et al. 2003; Blanke and Grusser 2001; Groh and Sparks 1996; Matsumiya 2022; Naffrechoux et al. 2024) but have not examined the time course of tactile localization during a saccade. In vision, a briefly flashed visual stimulus is mislocalized in the direction of a saccade when the visual stimulus is presented in the dark near the time of the saccade (Dassonville et al. 1995; Honda 1989; Mateeff 1978; Matin et al. 1969). This mislocalization typically appears about 100 ms before the saccade onset, peaks at the onset, and continues until approximately 100 ms afterward (Honda 1991). Furthermore, visual mislocalization also depends on the direction of the saccade. Indeed, leftward and rightward saccades produce opposite shifts in perceived visual location (Matin et al. 1970). However, it remains unknown whether a tactile stimulus briefly presented during a saccade is mislocalized in a similar way to the time course of visual mislocalization during a saccade. Because the standard theory of multisensory integration assumes that a retinal reference frame serves as a single frame of reference for mapping spatial information from all sensory modalities, the time course of tactile localization during a saccade would be expected to resemble that of visual localization during a saccade.

In this study, we investigated the time course of tactile localization during saccadic eye movements (Experiments 1 and 2: saccade condition). In Experiment 1, we also examined tactile localization under fixation conditions to evaluate whether gaze position alone (left vs. right) modulates perceived tactile location. Based on previous findings (Harrar and Harris 2009), we hypothesized that tactile localization would differ between left and right gaze positions even in the absence of saccades. To test this hypothesis, we performed a paired *t*-test comparing tactile localization between the two gaze positions. In addition, we hypothesized that tactile localization would vary systematically depending on the timing of the tactile stimulus relative to the saccade onset, and that this time course would be further modulated by the direction of the saccade. To test this hypothesis, we conducted a two-way repeated-measures analysis of variance (ANOVA) with stimulus timing and saccade direction as within-subject factors. Specifically, we expect that saccade direction reverses the sign of the localization bias, such that leftward and rightward saccades produce opposite shifts in perceived tactile position. Consequently, the main effect of stimulus timing may not reach significance when averaged across directions, due to cancellation of opposing biases. Therefore, a significant interaction between stimulus timing and saccade direction is essential for rejecting the null hypothesis. To further explore this interaction, we conducted simple main effect analyses for each saccade direction. This allowed us to examine whether tactile localization varied across stimulus timing within each saccade direction.

We also examined the effect of horizontal saccade direction on tactile localization in the depth dimension (Experiment 1). Previous studies in visual localization have shown that saccades cause mislocalization only in the direction of the saccade (Dassonville et al. 1995; Honda 1989, 1991; Matin et al. 1970). If horizontal saccades also affect tactile localization in the depth direction, then this effect should be observable. To test this, we conducted a two-way repeated-measures ANOVA with stimulus timing and saccade direction as within-subject factors.

Furthermore, we investigated whether attention contributes to tactile localization in the absence of saccades (Experiment 3: attention condition), in which participants maintained fixation. This investigation was motivated by previous findings that attention can modulate perceived tactile location (Flach and Haggard 2006; Kilgard and Merzenich 1995), raising the possibility that perceptual tactile localization during saccades may be driven by attention directed toward the saccade target (Deubel and Schneider 1996). However, other studies have shown that eye position itself can exert a stronger influence on tactile localization than attention alone (Harrar and Harris 2009), suggesting that oculomotor signals may play a more dominant role. Therefore, we hypothesized that the time course of tactile localization in the attention condition would differ from that in the saccade condition. To test this hypothesis, we examined whether tactile localization varied as a function of the timing of the tactile stimulus relative to the onset of an attentional cue that instructed participants to covertly attend to a lateral position without making any saccade, and compared the temporal profiles of tactile localization between the attention condition (Experiment 3) and the saccade condition (Experiment 2) using a three-way mixed-design ANOVA. The ANOVA included stimulus timing and cue or saccade direction as within-subject factors, and condition as a between-subject factor.

## General Methods

### Participants

Participants were recruited from a paid participant pool (Sona Systems, Ltd.) and received a gift card of 1,000 Japanese yen per hour for their participation. This paid participant pool comprised individuals who wished to participate in research studies being conducted by Tohoku University faculty members and graduate students. Undergraduate and graduate students of Tohoku University were among those registered in the paid participant pool. All participants were right-handed and had normal or corrected-to-normal vision. They provided written informed consent for participation, in accordance with the Code of Ethics of the World Medical Association (Declaration of Helsinki). The study was approved by the Ethics Committee of the Graduate School of Information Sciences, Tohoku University (approval no. 83(4–6)). All methods were conducted in accordance with relevant guidelines and regulations.

### Apparatus and Stimuli

**Figure 1.**
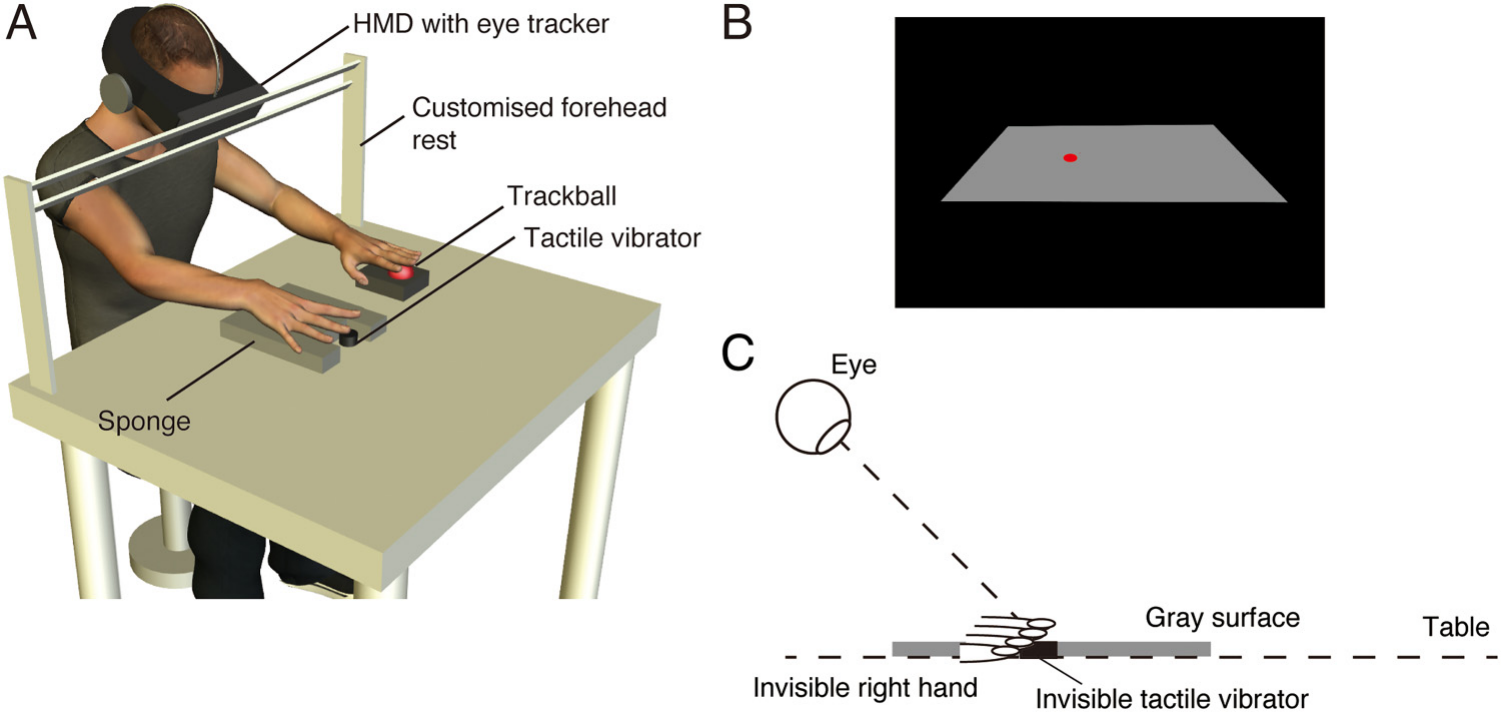
Apparatus and stimuli. (A) Participants binocularly viewed a visual stimulus presented through a head-mounted display and placed the middle finger of their right hand on a tactile vibrator. (B) A red fixation point was presented on a gray surface in the virtual environment. (C) The gray surface was spatially aligned with a real table, where the participant's right hand and the tactile vibrator were placed out of view. The participant's eyes were directed to the gray surface.

A tactile vibrator (Vibro Transducer Vp216; Acouve Laboratory, Inc., Tokyo, Japan) was used to present tactile stimuli. The Vp216 is a device that converts electrical audio signals into mechanical vibrations, functioning similarly to a speaker but specifically designed for tactile acoustic applications. In our experiments, the tactile stimuli were clearly perceptible but not uncomfortable. Participants generally described the tactile sensations as light to moderate in intensity. The tactile vibration was a sinusoidal tactile acoustic vibration with a temporal frequency of 100 Hz, which was presented for 50 ms to the ventral side of the tip of the middle finger of the participant's right hand. The tactile vibrator was equipped with a flat sponge that was positioned on a table (Figure 1A). The tip of the middle finger of the participant's right hand was placed on the tactile vibrator, and their whole hand was placed on the sponge. Participants listened to white noise presented through headphones (ATH-S600; Audio-Technica, Tokyo, Japan) to mask surrounding auditory input that could have provided additional cues about the location of the tactile stimulus.

**Figure 2.**
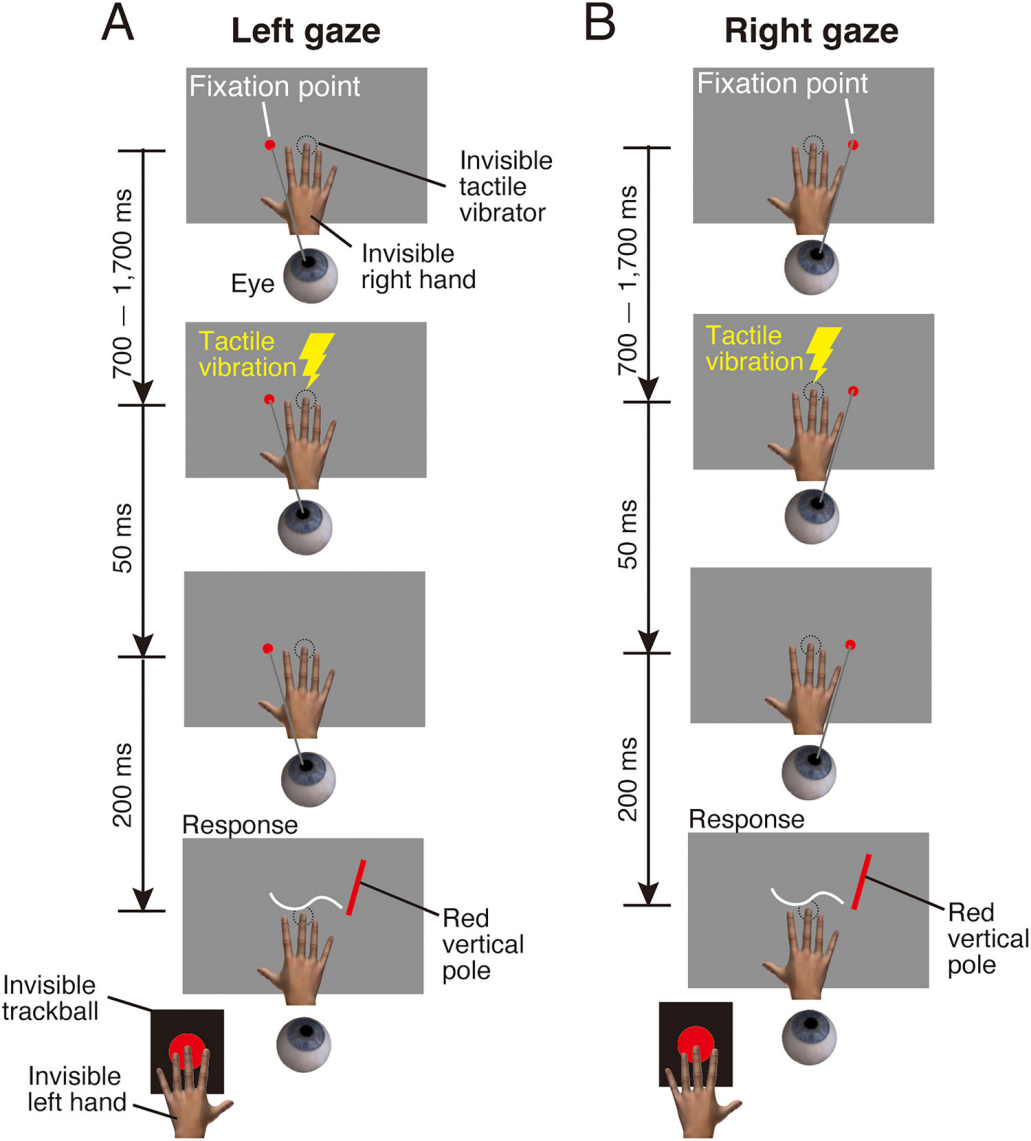
Stimulus sequence in the fixation condition of Experiment 1. (A) Left gaze. (B) Right gaze. Participants kept fixating on a fixation point. After a delay selected randomly to be between 700 and 1,700 ms, the tactile stimulus was presented for 50 ms. The red vertical pole appeared 200 ms after the tactile stimulus and the fixation point disappeared. The participant localized the perceived position of the tactile stimulus by moving the red vertical pole with a trackball.

**Figure 3.**
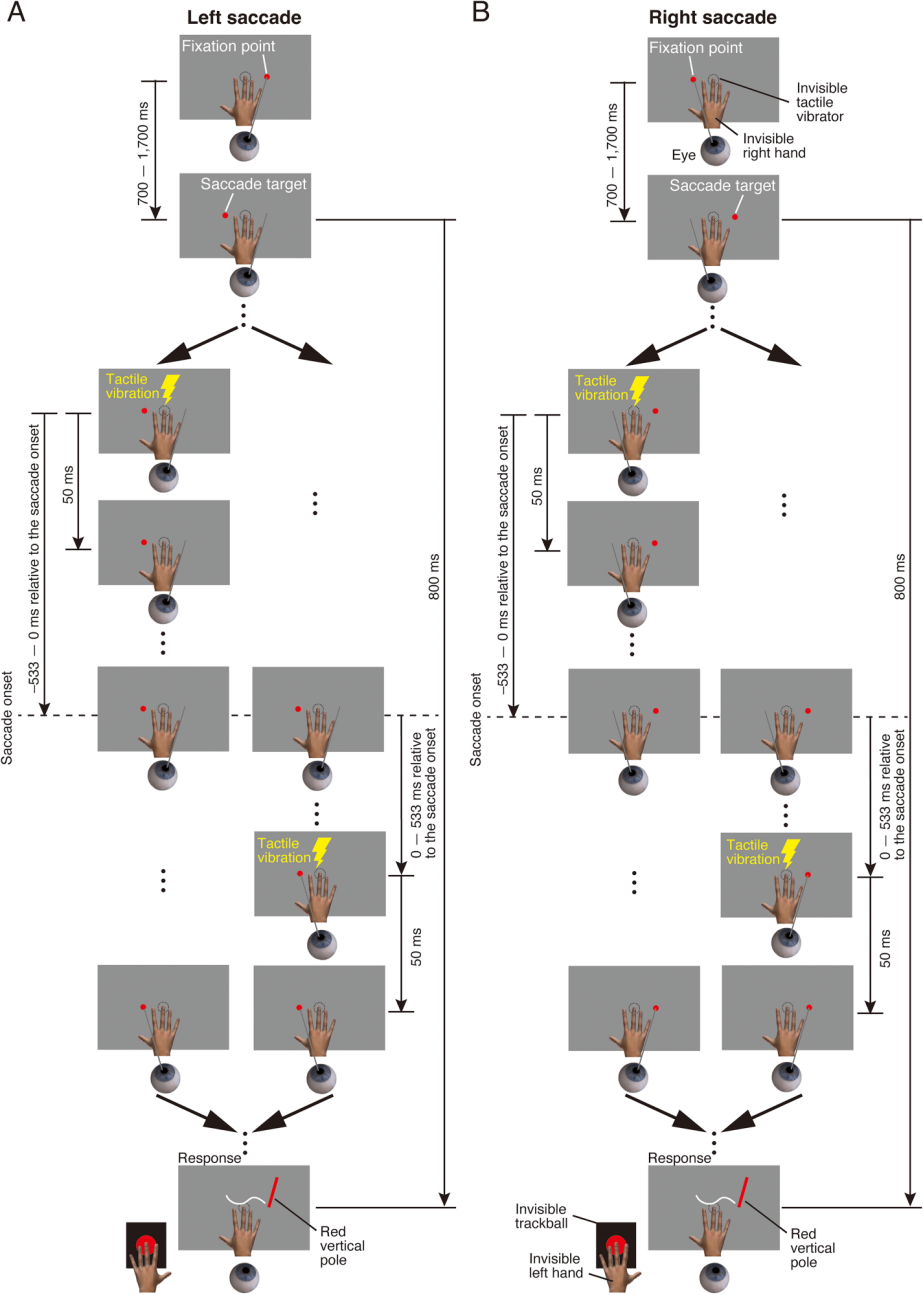
Stimulus sequence in the eye-movement condition of Experiment 1. (A) Left saccade. (B) Right saccade. Participants maintained fixation on a fixation point. After a randomly selected delay between 700 and 1,700 ms, the fixation point disappeared and the saccade target appeared. Participants were instructed to make a saccade toward the target as quickly as possible. A tactile stimulus was then presented for 50 ms. The timing of the tactile stimulus presentation relative to saccade onset was randomly selected from 13 time points: ±533, ±444, ±356, ±267, ±178, ±89, and 0 ms. After the saccade target disappeared, a red vertical pole appeared. Participants localized the perceived position of the tactile stimulus by moving the red vertical pole using a trackball.

Participants also wore a head-mounted display (HMD; Tobii VR Integration based on the HTC Vive, comprising dual 3.6-inch microdisplays with 1080 × 1200 pixels per eye and a 110° diagonal field-of-view; Tobii Technology, Stockholm, Sweden) that displayed visual stimuli (Tachibana and Matsumiya 2022). The HMD was equipped with an eye tracker that measured the gaze position of the participant's eye at a sampling rate of 120 Hz. The simulated viewing distance was 45 cm in virtual space. The HMD was covered with black tissue to occlude all surrounding visual input and was fixed to a customized forehead rest. This setup ensured that the participant's head position remained stable throughout the experiment, effectively minimizing head movements and allowing for consistent alignment between the visual stimuli and the participant's viewpoint. Participants viewed a gray surface (76° × 48°, 26.2 cd/m^2^) through the HMD (Figures 1B and C) and could not see their hands or the tactile vibrator. The gray surface was spatially aligned with the real table, where the participant's hand and the tactile vibrator were placed. A red fixation point (0.4° in diameter, 15.4 cd/m^2^) and a saccade target (0.4° in diameter, 15.4 cd/m^2^) were presented on the gray surface. The positions of the fixation point and saccade target differed between experiments. In Experiment 1, both the fixation point and the saccade target were located either 10° to the left or 10° to the right of the center of the gray surface (see Figures 2 and 3). In Experiments 2 and 3, the fixation point was located at the center (0°) of the gray surface. In Experiment 2, the saccade target appeared either 12° to the left or 12° to the right of the center of the gray surface (see Figure 9). In Experiment 3, the attentional cue appeared either 12° to the left or 12° to the right of the center of the gray surface (see Figure 11).

### Procedures

Two conditions were used in Experiments 1 and 2: a fixation condition and an eye-movement condition. In the fixation condition, participants were instructed to keep fixating on a fixation point (Figure 2). The tactile vibrator was attached to the ventral surface of the distal phalanx of the participant's right middle finger, which rested on a fixed position on the table throughout the experiment. To prevent the transmission of vibrations to the table, a sponge was placed between the tactile vibrator and the table surface. This setup minimized the propagation of tactile vibrations through the table, thereby reducing potential uncertainty about stimulus location and the generation of sound. Additionally, participants wore headphones and listened to white noise throughout the experiment to mask any auditory cues associated with the tactile stimulation. Each block began with an eye movement calibration procedure during which the participant fixated on 10 dots presented sequentially along the horizontal and depth center lines of the visual display and pressed a button after each fixation was completed. While participants were gazing at the fixation point, they pressed a button to start a trial. After a delay selected randomly to be between 700 and 1,700 ms, the tactile stimulus was presented for 50 ms. A red vertical pole (1.3° in diameter and 3.8° in length, 15.4 cd/m^2^) was presented in the virtual space 200 ms after the tactile stimulus and the fixation point disappeared. The red vertical pole was presented on the gray surface that was spatially aligned with the real table where the tactile vibrator was placed (see Figure 2). The tactile vibrator was located at the center of the gray surface. On each trial, the red vertical pole appeared at one of three possible locations, randomly selected: 20° to the right of the center, 20° to the left of the center, or 20° behind the center of the gray surface. Due to this large spatial separation between the red vertical pole and the tactile vibrator, we consider the likelihood of perceptual integration between these stimuli to be minimal. The participant localized the perceived position of the tactile stimulus by moving the red vertical pole horizontally and in depth on the gray surface with a trackball, allowing us to analyze tactile localization in both horizontal and depth dimensions. The trackball was used for the localization task instead of hand movements that point to the felt position of the tactile stimulus. Previous studies have reported that hand movements point to the veridical, rather than the felt, position of a tactile stimulus even when the perceived position is shifted from the physical one (Dijkerman and de Haan 2007; Kammers et al. 2009; Marcel 2003), suggesting that hand movements may not be suitable for investigating tactile perceptual localization. Since our task required participants to make a perceptual judgment about the location of a tactile stimulus, we considered the trackball to be a more appropriate tool for capturing tactile perceptual localization. The participant's gaze was monitored throughout all trials. Trials in which their gaze was not maintained within ±2° of the initial fixation position were considered invalid. However, all trials met this criterion.

In the eye-movement condition, participants were instructed to make a saccade to the right or left (Figure 3 for Experiment 1; Figure 9 for Experiment 2). Each block began with an eye movement calibration procedure in which the participant fixated on 10 dots presented sequentially along the horizontal and depth center lines of the visual display and pressed a button after each fixation was completed. Then, the participant fixated on the fixation point and pressed a button to start a trial. After a randomly selected delay of between 700 and 1,700 ms, the fixation point disappeared and the saccade target appeared. The participant made a saccade toward the saccade target as quickly as possible. The tactile stimulus was presented for 50 ms. The timing of the tactile stimulus presentation relative to saccade onset was randomly selected from 13 timings: ±533, ±444, ±356, ±267, ±178, ±89, and 0 ms. To efficiently schedule the tactile stimulus presentation—particularly for trials intended to present the tactile stimulus before saccade onset—the timing was determined based on the averaged saccade onset time over the past four trials (Matsumiya and Uchikawa 2001, 2003). Specifically, saccade onset time was defined as the moment when the eye began to move following the disappearance of the fixation point and the presentation of the saccade target (see the *Analysis of eye movements* section for details). After each trial, the actual saccade onset time was recorded, and the average of the most recent four trials was used to estimate the expected saccade onset time for the next trial. In the first four trials of each block, the estimated saccade onset was set to 200 ms after the onset of the saccade target. This fixed value was used to allow the initial trials to proceed before saccade onset could be estimated based on the participant's actual eye movement data. The tactile stimulus presentation was then scheduled relative to this estimated saccade onset time. For example, if the estimated saccade onset was 250 ms and the tactile stimulus was intended to be presented 178 ms before saccade onset, the tactile stimulus was presented 72 ms after the disappearance of the fixation point and the presentation of the saccade target. In another example, if the estimated saccade onset was 250 ms and the tactile stimulus was intended to be presented 89 ms after saccade onset, the tactile stimulus was presented 339 ms after the disappearance of the fixation point and the presentation of the saccade target. Importantly, the estimated saccade onset time was used only during the experiment and was not recorded or used in any data analysis. For data analysis, the actual saccade onset time was measured on each trial and used to determine the precise timing of the tactile stimulus relative to saccade onset, even when the actual saccade onset time differed from the estimated one. This approach allowed for accurate temporal alignment between stimulus presentation and saccade onset. Both the disappearance of the saccade target and the presentation of a red vertical pole occurred 800 ms after the presentation of the saccade target. The timing of the red vertical pole presentation was locked to the cue, not to the actual onset of the saccade. Although the presentation of the red vertical pole was not synchronized with real-time eye movement data, we monitored the timing of the saccade. Trials in which the actual saccade onset occurred later than 600 ms after the cue were considered invalid. This criterion ensured that, in valid trials, the red vertical pole was presented at a time point that typically followed the saccade. The participant localized the perceived position of the tactile stimulus by moving the red vertical pole with a trackball. After moving the pole to the perceived location of the tactile stimulus, the participant pressed a button to indicate their chosen localization position.

Trials were repeated during the experiment until a valid trial was obtained if any of the following criteria were not met: saccade latency was shorter than 100 ms or longer than 600 ms, a saccade was made before the saccade target was presented, a saccade was made in the opposite direction to the saccade target, or the horizontal saccade amplitude was less than 5°. These failed trials were not repeated immediately after the error occurred. Instead, they were randomly reinserted later within the same block to maintain the natural flow of the experiment and to minimize participants’ awareness of the repetition. Because failed trials were repeated until a valid trial was obtained, their number was not counted or recorded.

**Figure 4.**
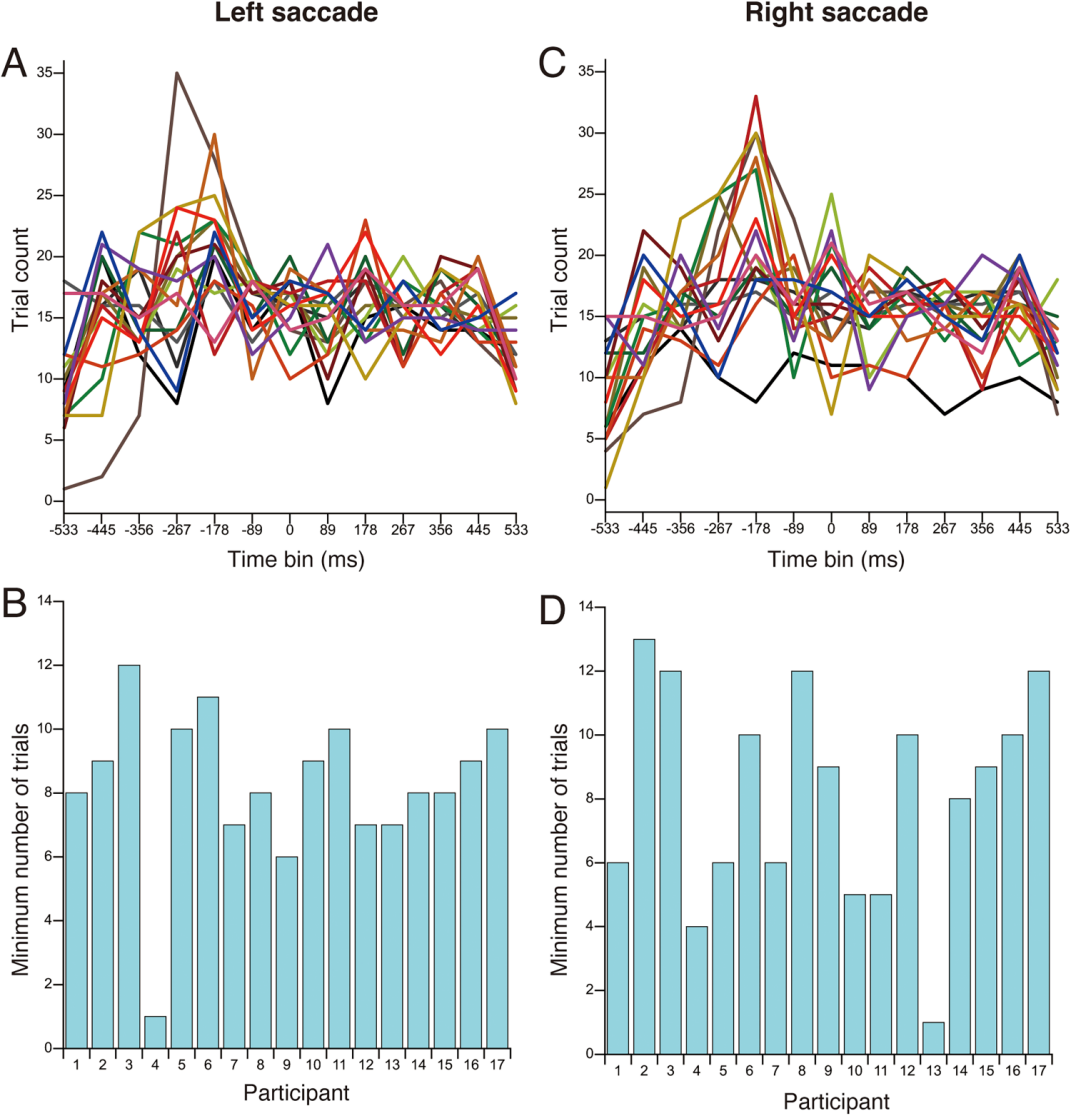
The number of trials included in each time bin and the minimum number of trials per participant for Experiment 1. (A) Number of trials as a function of time bin for left saccade. (B) Minimum number of trials per participant for left saccade. (C) Number of trials as a function of time bin for right saccade. (D) Minimum number of trials per participant for right saccade. Each colored line represents a different participant.

**Figure 5.**
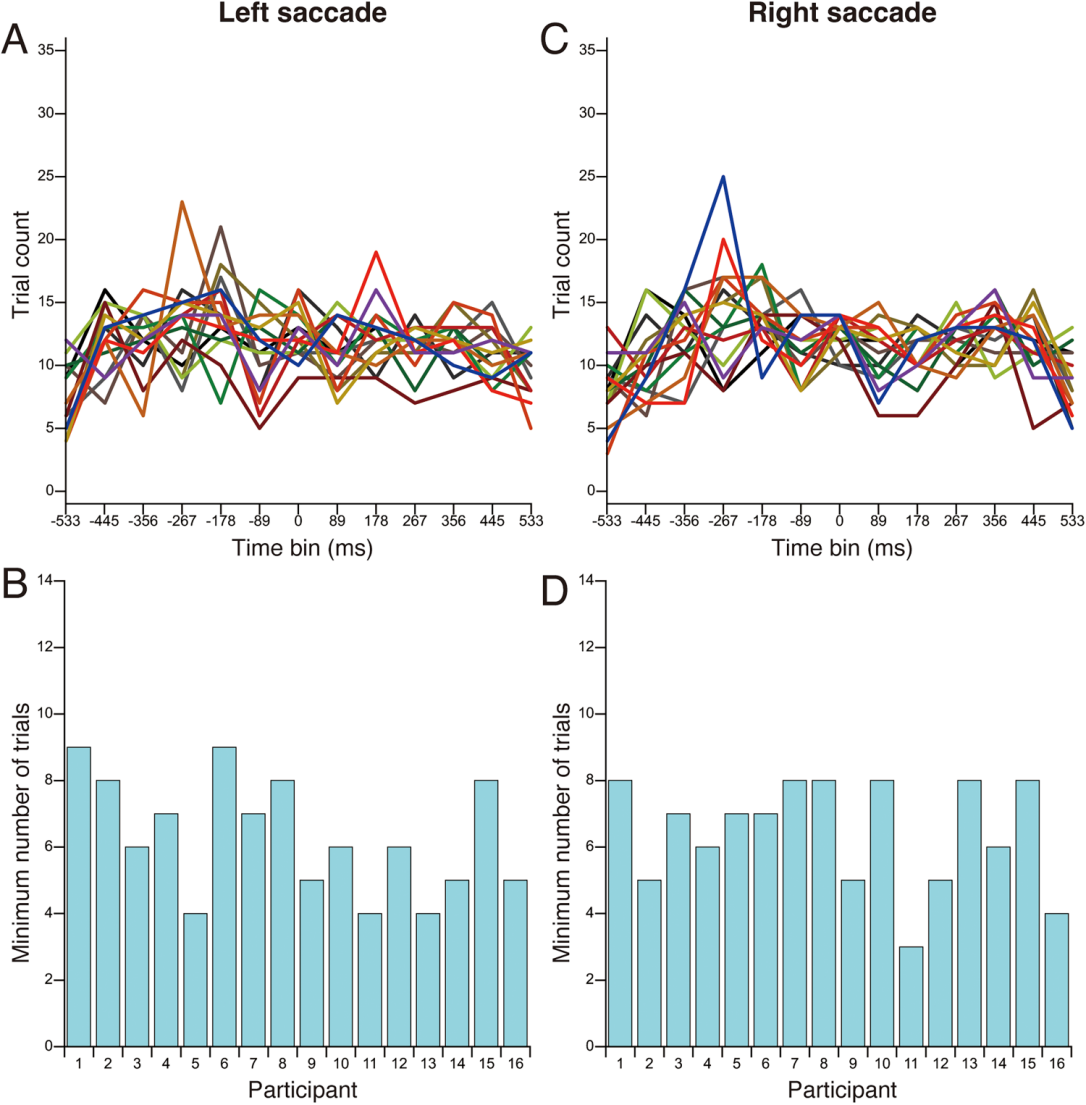
The number of trials included in each time bin and the minimum number of trials per participant for Experiment 2. (A) Number of trials as a function of time bin for left saccade. (B) Minimum number of trials per participant for left saccade. (C) Number of trials as a function of time bin for right saccade. (D) Minimum number of trials per participant for right saccade. Each colored line represents a different participant.

Trials in which the timing of the tactile stimulus presentation did not fall within the range of −533 ms to +533 ms relative to the actual saccade onset were excluded from the analysis in both Experiments 1 and 2. No data were excluded from the analysis in Experiment 3. In Experiments 1 and 2, the timing of the tactile stimulus was determined based on the estimated saccade onset; therefore, discrepancies between the estimated and actual saccade onset occasionally caused the tactile stimulus to fall outside the −533 ms to +533 ms range relative to the actual saccade onset. In Experiment 1, 296 trials were excluded from a total of 7,072 trials across all participants (4.2%). In Experiment 2, 195 trials were excluded from a total of 4,992 trials (3.9%). Figures 4 and 5 show the number of trials included in each time-bin and the minimum number of trials per participant for Experiments 1 and 2, respectively. These results indicate that a small number of participants had time-bins with an extremely low number of trials. Based on these findings, data from time-bins with fewer than four trials were excluded from the analysis. Only participants with complete data across all time-bins were included in the statistical analysis. In Experiment 1, two participants were completely excluded from the analysis due to missing data in one or more time-bins. One participant lacked valid data in the −533 ms and −444 ms time-bins in the left saccade condition, and the other participant lacked valid data in −533 ms time-bin in the right saccade condition. In Experiment 2, one participant was completely excluded from the analysis due to missing data in the −533 ms time-bin in the right saccade condition. To ensure consistency between the statistical analysis and the visual representation of the data, these participants were also excluded from the figures.

In each trial, a single tactile stimulus was presented to the same physical location on the finger. Prior to the experiment, participants were explicitly instructed not to rely on memory of the physical stimulus location or on hand posture when reporting the perceived location. Instead, they were asked to report the subjectively perceived location of the tactile stimulus on each trial. This instruction was emphasized to ensure that the task measured tactile localization rather than memory-based reproduction or posture-based estimation.

### Assessment of Tactile Localization

**Figure 6.**
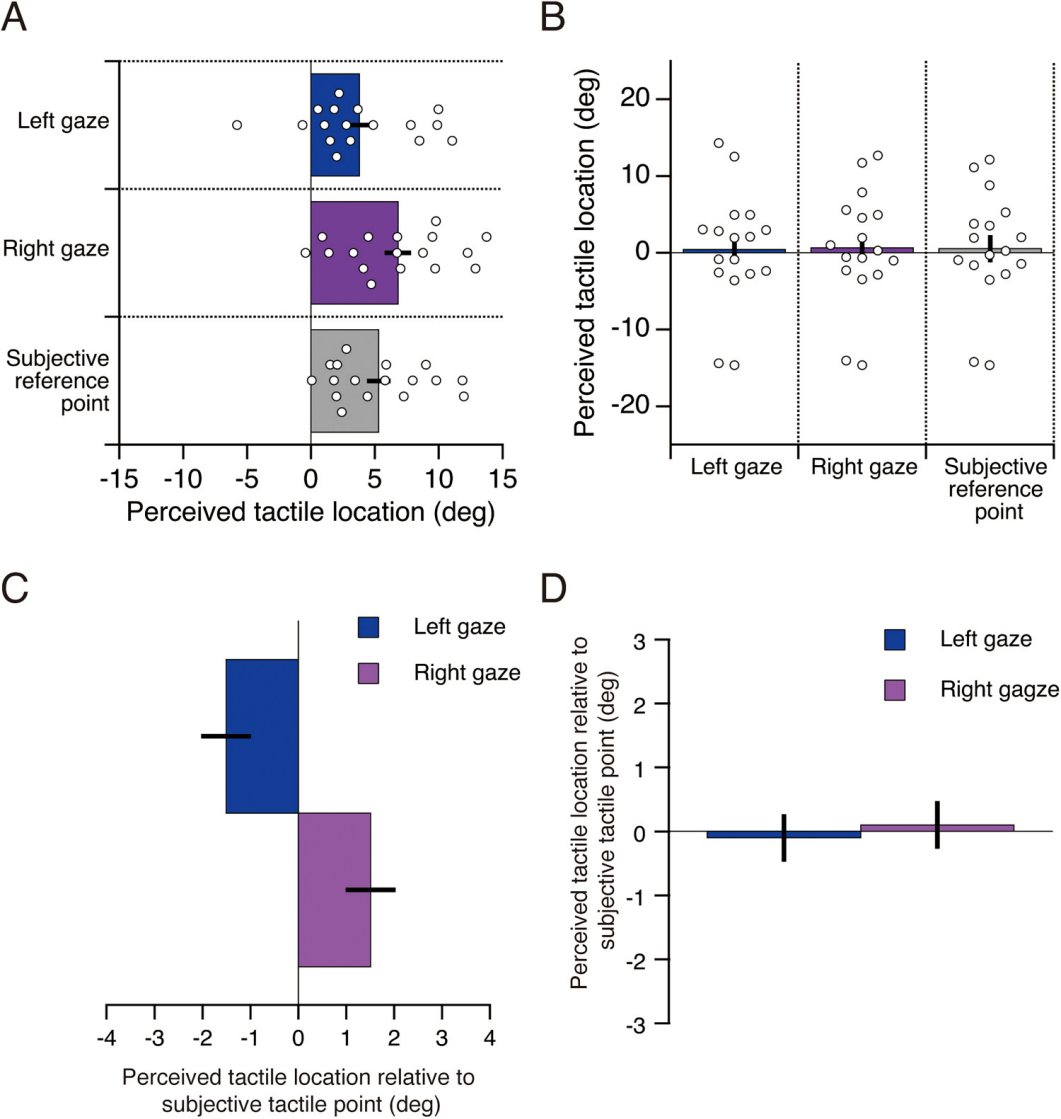
Perceived horizontal and depth locations of tactile stimuli when participants gazed at a fixation point in Experiment 1. (A) Horizontal tactile locations under left and right gaze conditions. (B) Depth tactile locations under left and right gaze conditions. Panels A and B illustrate individual perceived tactile locations. The subjective reference point was determined as the mean perceived tactile locations under the left and right gaze conditions for each participant. Each open symbol represents a different participant, and the colored bars represent the mean perceived tactile locations. (C) Horizontal tactile locations expressed as deviations from the subjective reference point. (D) Depth tactile locations expressed as deviations from the subjective reference point. Panels C and D illustrate normalized tactile localization relative to each participant's subjective reference point. Positive values of the perceived horizontal tactile location indicate a right side relative to the subjective reference point. Positive values of the perceived depth tactile location indicate an upper side relative to the subjective reference point. The dark blue and purple bars represent the left and right gazes, respectively. Results are the mean ± standard error.

A normalization procedure was applied consistently across all three experiments to allow comparison of tactile localization relative to each participant's own perceptual baseline. Given the large individual differences in absolute tactile localization (Figures 6A and B), we defined a subjective reference point for each participant as the mean perceived tactile location obtained during the fixation condition. Tactile localization was then calculated as deviations from this subjective reference point. This approach enabled us to normalize tactile spatial perception.

In Experiment 1, two fixation conditions were used: left fixation and right fixation. The subjective reference point was defined as the average of the mean perceived tactile locations obtained during these two fixation conditions. Tactile localization was assessed in both horizontal (left–right) and depth (near–far) dimensions and analyzed separately for both the fixation and eye-movement conditions.

In Experiments 2 and 3, only a single fixation condition was used, in which participants fixated at the center of the gray surface. In these experiments, the subjective reference point was defined as the mean perceived tactile location obtained during this central fixation condition, and only horizontal tactile localization was evaluated.

### Analysis of Eye Movements

**Figure 7.**
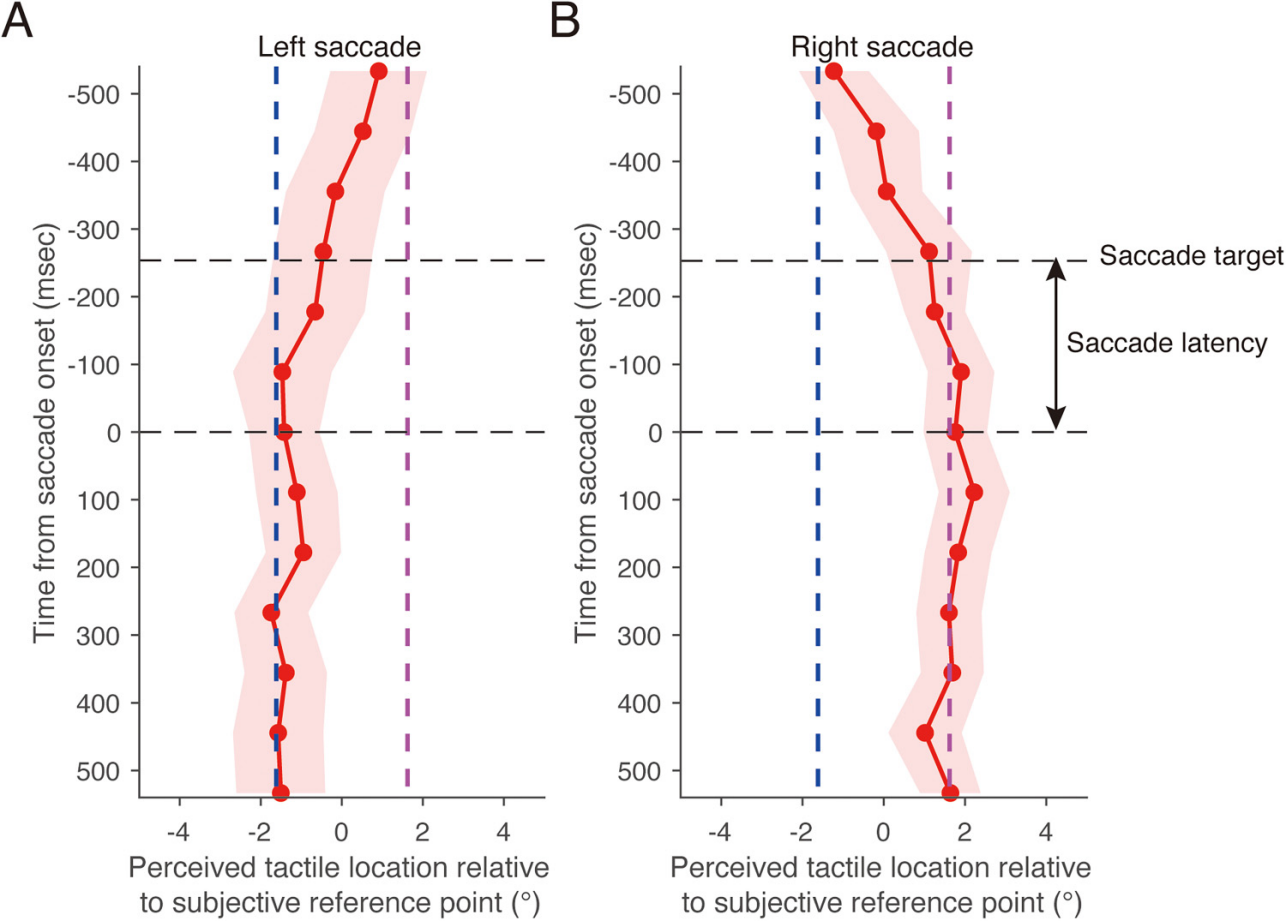
Perceived horizontal location of tactile stimuli during a saccade as a function of time from saccade onset in Experiment 1. (A) Left saccade. (B) Right saccade. Positive values of the perceived tactile location indicate a right side relative to the subjective reference point. The dark blue dashed line represents the horizontal location that was perceived when the left fixation point was gazed at during the fixation condition. The purple dashed line represents the perceived horizontal location when the right fixation point was gazed at during the fixation condition. The black dashed line labeled “saccade target” represents the mean timing of the saccade target presentation. Results are the mean ± standard error.

**Figure 8.**
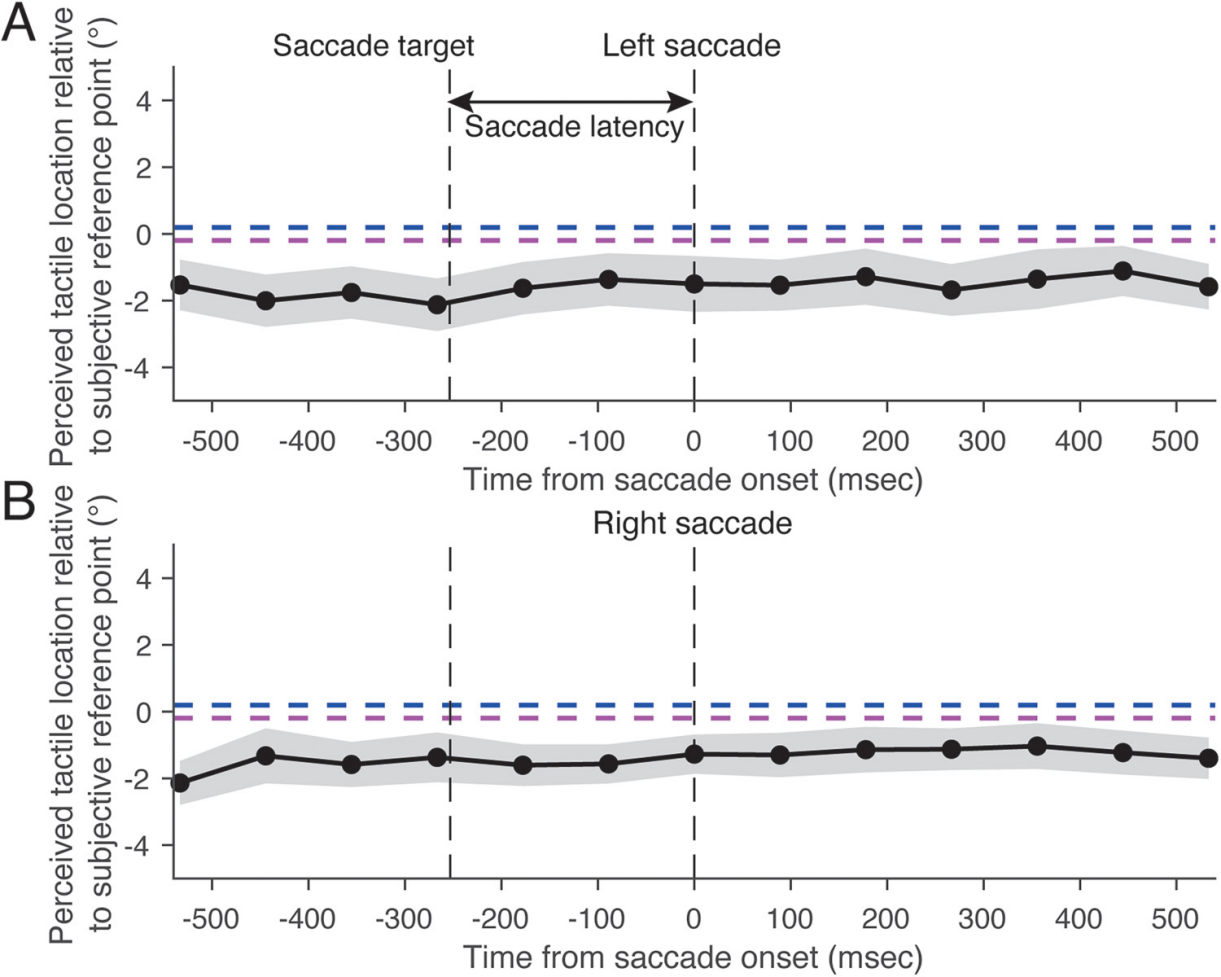
Perceived depth location of tactile stimuli during a saccade as a function of time from saccade onset in Experiment 1. (A) Left saccade. (B) Right saccade. Positive values of the perceived tactile location indicate an upper side relative to the subjective reference point. The dark blue dashed line represents the perceived depth location when the left fixation point was gazed at during the fixation condition. The purple dashed line represents the perceived depth location when the right fixation point was gazed at during the fixation condition. The black dashed line labeled “saccade target” represents the mean timing of the saccade target presentation. Results are the mean ± standard error.

Saccade onset was defined as the time at which eye velocity exceeded 50°/s. This criterion is generally similar to those employed in previous studies (Deubel et al. 1996; Matsumiya and Uchikawa 2001, 2003), but was slightly adjusted to suit the characteristics of the eye tracker used in this study (i.e., the Tobii VR Integration system). The adjustment was based on pilot data and aimed to ensure accurate and reliable detection of saccade onset under our specific recording conditions. Four samples were used to calculate the eye velocity in each video frame to be displayed on the HMD with a 90-Hz refresh rate (Matsumiya 2021, 2022). Eye velocity was calculated based on the following formula (Usui and Amidror 1982):

$$v_{k}=\frac{x_{k+2}+x_{k+1}-x_{k-1}-x_{k-2}}{6T},\;k\;\geq 3$$

where *v_k_* is the eye velocity, *x_k+2_*, *x_k+1_*, *x_k−1_*, and *x_k−2_* are the eye positions, *T* is the sampling interval, and *k* is the frame index. This formula is derived by combining the digital first-order differentiation method with the moving average method, resulting in a velocity estimate that is effectively low-pass filtered. In each trial, saccade latency was defined as the period between the target onset and the saccade onset. The saccade latency shown in Figures 7, 8, and 10 represent the mean across all trials.

### Statistical Analyses

To evaluate our hypothesis in the fixation condition of Experiment 1, we performed a paired *t*-test comparing tactile localization between two gaze conditions (left gaze and right gaze). A statistically significant difference between these conditions would lead to the rejection of the null hypothesis, which posits that gaze position does not modulate tactile localization.

To evaluate our hypotheses in the eye-movement condition of Experiments 1 and 2, we conducted a two-way repeated-measures ANOVA with stimulus timing (13 time bins) and saccade direction (left vs. right) as within-subject factors. The null hypothesis states that tactile localization does not differ across saccade directions or stimulus timings. In our design, however, saccade direction is expected to reverse the sign of the localization bias (i.e., leftward and rightward saccades produce opposite shifts in perceived tactile location). As a result, the main effect of stimulus timing may not reach significance when data are averaged across saccade directions, due to cancellation of opposing biases. Therefore, a significant interaction between stimulus timing and saccade direction is critical for rejecting the null hypothesis. This interaction would indicate that tactile localization is modulated by both the timing of the stimulus relative to the saccade onset and the direction of the saccade. To further investigate the interaction, we conducted simple main effect analyses for each saccade direction.

To further compare the temporal profiles of tactile localization between the attention and saccade condition (Experiment 3), we conducted a three-way mixed-design ANOVA with stimulus timing (13 time bins) and cue or saccade direction (left vs. right) as within-subject factors, and condition (attention vs. saccade) as a between-subject factor. This analysis was designed to test whether attentional shifts alone without accompanying saccadic eye movements (Experiment 3) could account for perceptual tactile localization observed in the saccade condition (Experiment 2). A significant interaction between condition and cue or saccade direction would indicate that the effect of cue direction (in the attention condition) or saccade direction (in the saccade condition) on perceived tactile location differs between the two conditions. In other words, the pattern of tactile localization for leftward versus rightward direction is modulated by whether participants were attending to a cue or executing a saccade, suggesting that attentional and oculomotor processes influence tactile spatial perception in distinct ways. To further interpret this interaction, we performed simple main effect analyses separately for the attention and saccade conditions. Data were processed in MATLAB R2024a (MathWorks Inc., Natick, MA) and analyzed using SPSS version 29 (IBM Corp., Armonk, NY).

## Experiment 1

### Methods

A total of 17 participants (5 women and 12 men; mean age, 25.3 [range, 18–50] years) were recruited for Experiment 1. All participants were right-handed and had normal or corrected-to-normal vision.

In the fixation condition, participants were instructed to keep fixating at −10° (left fixation) or 10° (right fixation) from the center of the gray surface (see General Methods and Figure 2). These fixation points were randomly determined for each trial. Eye position was continuously monitored throughout the trials using the eye tracker. No eye movements were detected during these trials. Each participant performed 10 trials in total: 5 trials with left fixation and 5 trials with right fixation.

In the eye-movement condition, participants were instructed to make a saccade to the right or left (see General Methods and Figure 3). When making a saccade to the right, the fixation point was presented −10° (left side) from the center of the gray surface and the saccade target was presented 10° (right side) from the center of the gray surface. When making a saccade to the left, the fixation point was presented 10° (right side) from the center of the gray surface and the saccade target was presented −10° (left side) from the center of the gray surface. Each participant performed 8 blocks (2 saccade directions × 4 repetitions). Within each block, 52 trials (13 timings of the tactile stimulus × 4 repetitions) were conducted in a random order. As a result, each timing of the tactile stimulus was presented 16 times per saccade direction in total across all blocks (4 repetitions per block × 4 blocks). The saccade direction was fixed across trials in a block. The order of saccade directions was counterbalanced across participants. Short breaks were provided between each block.

### Results and Discussion

In the fixation condition, the perceived horizontal location of the tactile stimulus was shifted in the same direction as the eye position (Figure 6C), which is consistent with the results of a previous study (Harrar and Harris 2009). That is, when participants gazed to the left, they perceived the touch as being more toward the left than when they gazed to the right. There was a significant difference in perceived horizontal tactile locations between left and right gazes (*t_16_* = 2.90, *p* < .05, *d* = 0.70). In contrast, perceived depth tactile locations were not affected by eye position (Figure 6D). There was no significant difference in perceived depth tactile locations between left and right gazes (*t_16_* = 0.27, *p* = .79, *d* = 0.066). These results indicate that only the horizontal location of a tactile stimulus is biased by the direction of eye position.

Figure 7 shows the mean perceived horizontal tactile location as a function of time relative to the mean saccade onset in the eye-movement condition. A significant interaction was found between stimulus timing and saccade direction (*F*(12, 168) = 3.43, *p* < .01, *f* = 0.46), along with a marginally significant main effect of saccade direction (*F*(1, 14) = 3.46, *p* = .081, *f* = 0.46). There was no significant main effect of stimulus timing (*F*(12, 168) = 0.69, *p* = .76, *f* = 0.21), suggesting that the result may be due to the cancellation of opposing perceptual shifts when averaged across saccade directions. A simple main effect analysis revealed that the perceived horizontal tactile location during a leftward saccade significantly varied as a function of time relative to saccade onset (Figure 7A; *F*(12, 168) = 10.9, *p* < .01, *η^2^* = 0.23). Thus, 500 ms before the start of the leftward saccade (−500 ms in Figure 7A), the perceived horizontal location of the tactile stimulus shifted toward the location that was perceptually localized when the right fixation point was gazed at during the fixation condition (purple dashed line in Figure 7A), which was similar to the shift in perceived horizontal tactile location during the fixation condition. From −500 ms to −250 ms, the perceived horizontal tactile location gradually shifted toward the location that was perceptually localized when the left fixation point was gazed at during the fixation condition (dark blue dashed line in Figure 7A), although the saccade target was not presented in the display. After the saccade target was presented (−254 ms in Figure 7A; average saccade target presentation time across all trials), the perceived horizontal tactile location still shifted toward the location that was perceptually localized when the left fixation point was gazed at during the fixation condition. This perceptual shift continued until 500 ms after the saccade onset. A simple main effect analysis revealed that the perceived horizontal tactile location during a rightward saccade significantly varied as a function of time relative to saccade onset (Figure 7B; *F*(12, 168) = 13.7, *p* < .01, *η^2^* = 0.27). The time course of the horizontal tactile localization during a rightward saccade was almost the same as that during a leftward saccade, except for the direction of the shift in tactile localization being reversed.

In contrast, the perceived depth tactile location during a saccade did not depend on the time from the saccade onset for both left and right saccade directions (Figures 8A and B). There was no significant interaction between stimulus timing and saccade direction (*F*(12, 168) = 0.61, *p* = .83, *f* = 0.19), nor significant main effect of saccade direction (*F*(1, 14) = 0.07, *p* = .79, *f* = 0.067). Additionally, there was no significant main effect of stimulus timing (*F*(12, 168) = 1.74, *p* = .06, *f* = 0.33). These results suggest that tactile localization is not affected in the depth dimension when horizontal saccades are made.

Thus, these results indicate that tactile localization is biased only in the saccade direction and depends on the time from the saccade onset.

## Experiment 2

Experiment 1 showed that perceived horizontal tactile locations began to shift before the saccade target was presented. This suggests that participants may have formed a bias in tactile localization toward the direction of the saccade, because the saccade direction was fixed in the same direction during each block in Experiment 1. Indeed, it has been reported that repeated saccades in the same direction over several dozen trials cause mislocalization toward the saccade direction (Bahcall and Kowler 1999; Hopp and Fuchs 2004). In Experiment 2, to eliminate the bias caused by repeated saccades in the same direction, the saccade direction was randomized for each trial during each block. This experiment examined whether such a bias is required to generate the perceptual shift in tactile localization during a saccade.

### Methods

A total of 16 participants (5 women and 11 men; mean age 25.4 [range 19–50] years) were recruited for Experiment 2. All participants were right-handed and had normal or corrected-to-normal vision. Two of these participants also took part in Experiment 1.

**Figure 9.**
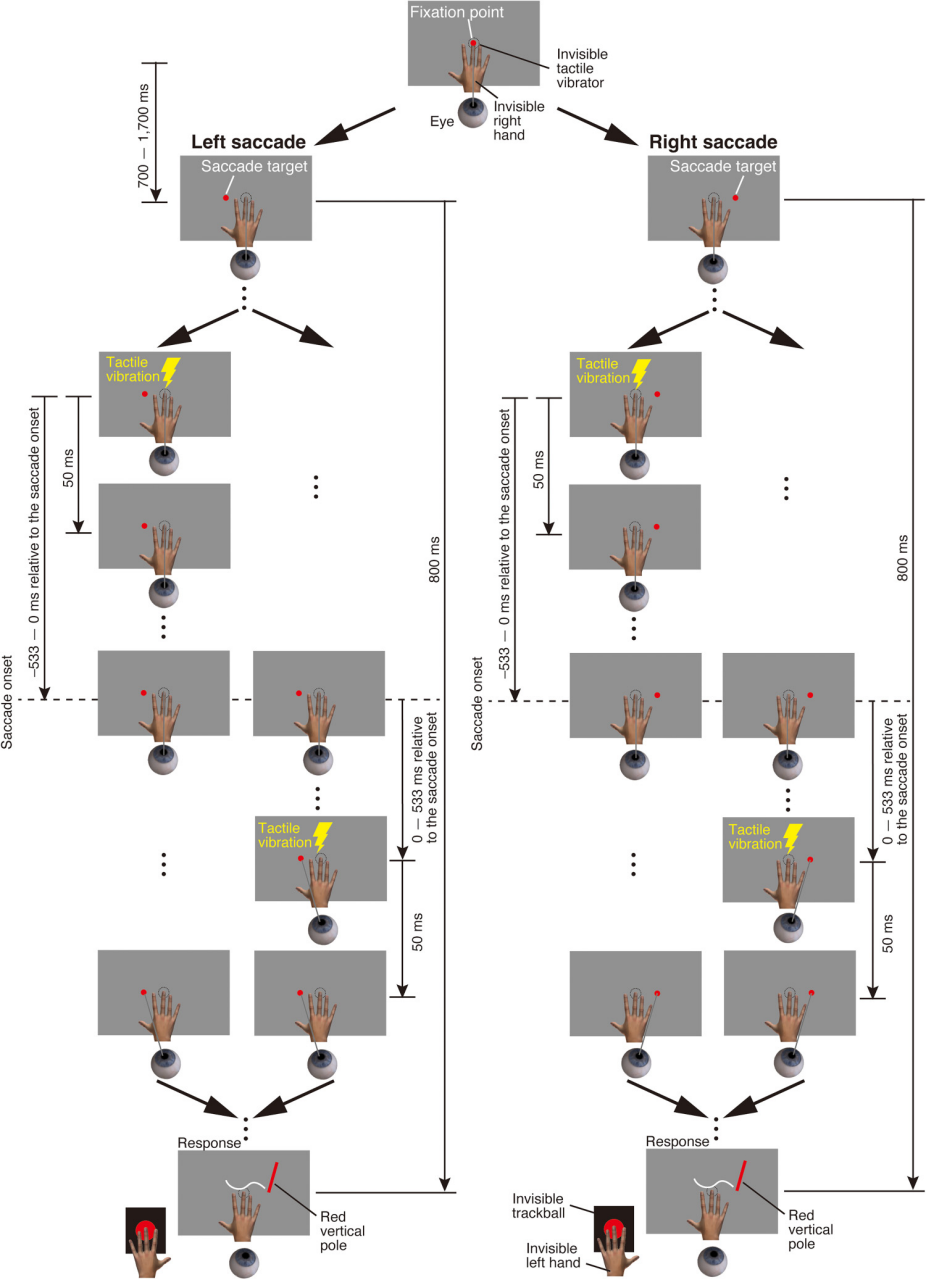
Stimulus sequence in the eye-movement condition of Experiment 2. Participants maintained fixation on a central fixation point. After a randomly selected delay between 700 and 1,700 ms, the fixation point disappeared and a saccade target appeared, either to the left or right of the fixation point. Participants were instructed to make a saccade toward the target as quickly as possible. A tactile stimulus was then presented for 50 ms. Its timing relative to saccade onset was randomly selected from 13 time points: ±533, ±444, ±356, ±267, ±178, ±89, and 0 ms. After the saccade target disappeared, a red vertical pole appeared. Participants localized the perceived position of the tactile stimulus by moving the red vertical pole using a trackball.

In the fixation condition, participants were instructed to keep fixating at 0° from the center of the gray surface (see General Methods and Figure 9). Eye position was continuously monitored throughout the trials using the eye tracker. No eye movements were detected during these trials. Each participant performed 5 trials in total.

In the eye-movement condition, the procedure of Experiment 2 was identical to that of Experiment 1 except in the following aspects (Figure 9). The fixation point was always presented at 0° from the center of the gray surface. The saccade target was randomly selected to be presented either −12° (left side) or 12° (right side) from the center of the gray surface for each trial. Each participant performed six blocks. Within each block, 52 trials (13 timings of the tactile stimulus × 2 saccade directions × 2 repetitions) were conducted in a random order. As a result, each timing of the tactile stimulus was presented 12 times per saccade direction in total across all blocks (2 repetitions per block × 6 blocks).

The analysis of the perceived tactile locations in Experiment 2 was identical to that in Experiment 1 except in the following aspects. First, the subjective reference point was defined as the perceived horizontal tactile location obtained during the fixation condition in Experiment 2 for each participant. Second, perceived depth tactile locations were not analyzed in Experiment 2.

### Results and Discussion

**Figure 10.**
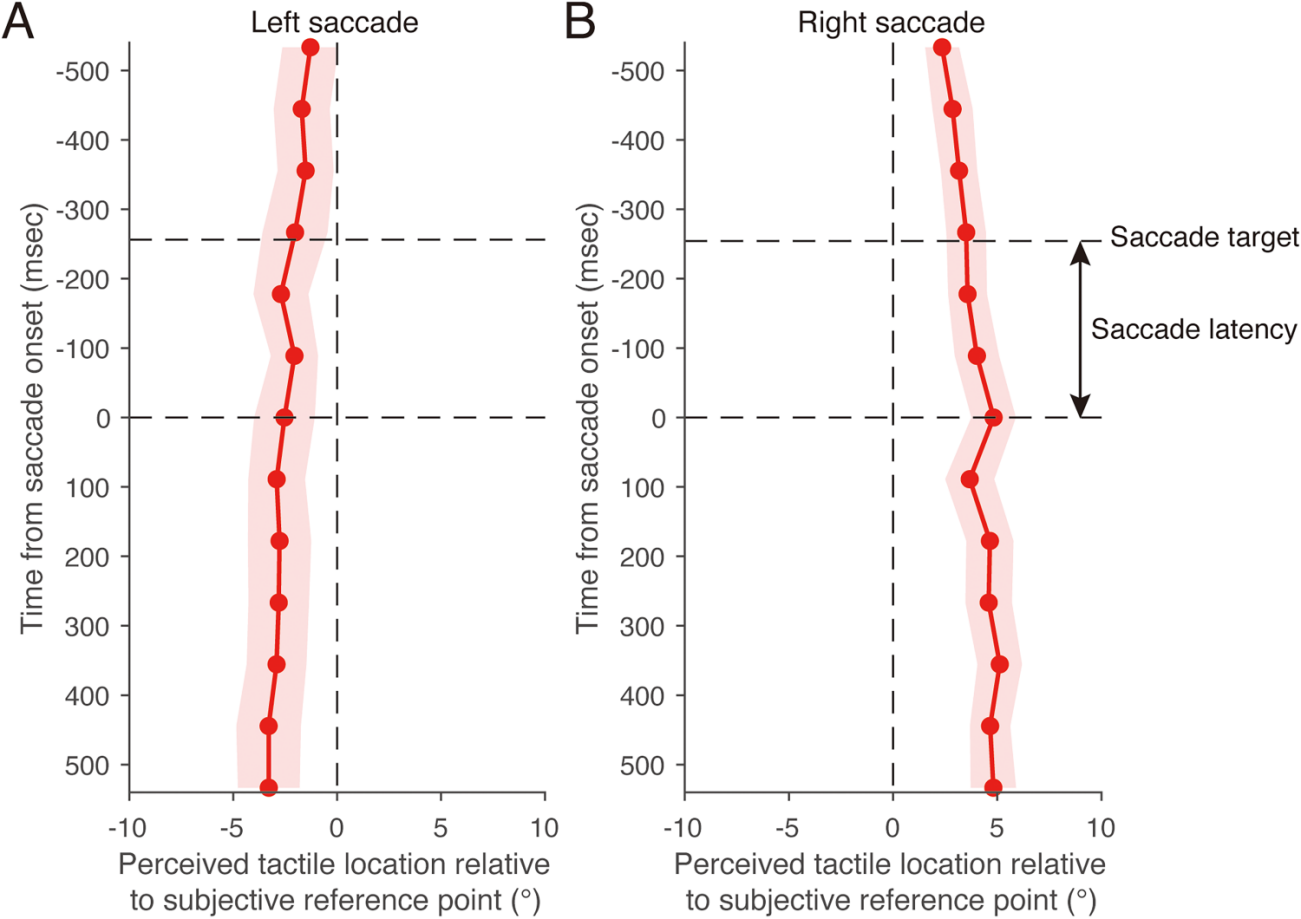
Perceived horizontal location of tactile stimuli during a saccade as a function of time from saccade onset in Experiment 2. (A) Left saccade. (B) Right saccade. Positive values of the perceived tactile location indicate a right side relative to the subjective reference point. The black dashed line labeled “saccade target” represents the mean timing of the saccade target presentation. Results are the mean ± standard error.

Figure 10 shows the mean perceived horizontal tactile location as a function of time relative to the mean saccade onset. A significant interaction was found between stimulus timing and saccade direction (*F*(12, 168) = 4.81, *p* < .01, *f* = 0.60), along with a significant main effect of saccade direction (*F*(1, 14) = 14.19, *p* < .01, *f* = 0.97). There was no significant main effect of stimulus timing (*F*(12, 168) = 0.80, *p* = .65, *f* = 0.23), suggesting that the result may be due to the cancellation of opposing perceptual shifts when averaged across saccade directions. A simple main effect analysis revealed that the perceived horizontal tactile location during a leftward saccade significantly varied as a function of time relative to saccade onset (Figure 10A; *F*(12, 168) = 3.36, *p* < .01, *η^2^* = 0.35). Thus, 500 ms before the start of the leftward saccade (−500 ms in Figure 10A), the perceived horizontal location of the tactile stimulus was near the location that was perceptually localized when the fixation point was gazed at during the fixation condition (the vertical dashed line in Figure 10A). From −500 ms to −250 ms, the perceived horizontal tactile location gradually shifted in the saccade direction, although the saccade target was not presented in the display. After the saccade target was presented (−256 ms in Figure 10A; average saccade target presentation time across all trials), the perceived horizontal tactile location still shifted in the saccade direction. This perceptual shift continued until 500 ms after the saccade onset. A simple main effect analysis revealed that the perceived horizontal tactile location during a rightward saccade significantly varied as a function of time relative to saccade onset (Figure 10B; *F*(12, 168) = 4.54, *p* < .01, *η^2^* = 0.40). The time course of the horizontal tactile localization during a rightward saccade was almost the same as that during a leftward saccade, except for the direction of the shift in tactile localization being reversed. These findings suggest that the tactile localization bias occurs independently of repeated saccade direction, even before the saccade target is presented.

## Experiment 3

Next, we examined the effects of attention on tactile localization. Previous studies have shown that perceived tactile locations shift with attention (Flach and Haggard 2006; Kilgard and Merzenich 1995). It is therefore possible that the perceptual shift in tactile localization toward the saccade target found in Experiments 1 and 2 might have been due to the redirection of attention toward the target. However, other studies have demonstrated that eye position can influence tactile localization more strongly than attention alone (Harrar and Harris 2009), suggesting that the time course of tactile localization in the attention condition may differ from that in the saccade condition. To test this possibility, we repeated Experiment 2 under an attention condition in which participants maintained fixation on the center of the gray surface without making saccades. We compared the temporal profiles of tactile localization between the attention condition (Experiment 3) and the saccade condition (Experiment 2). This comparison was designed to dissociate the effects of attention from those of eye position. By comparing these conditions, we aimed to determine whether the perceptual shift in tactile localization is primarily driven by attentional allocation or by saccadic eye movements.

### Methods

A total of 15 participants (5 women and 10 men; mean age 27.5 [range 19–50] years) were recruited for Experiment 3. All participants were right-handed and had normal or corrected-to-normal vision. One participant took part in all three experiments, one participated in both Experiments 1 and 3, and another participated in both Experiments 2 and 3.

**Figure 11.**
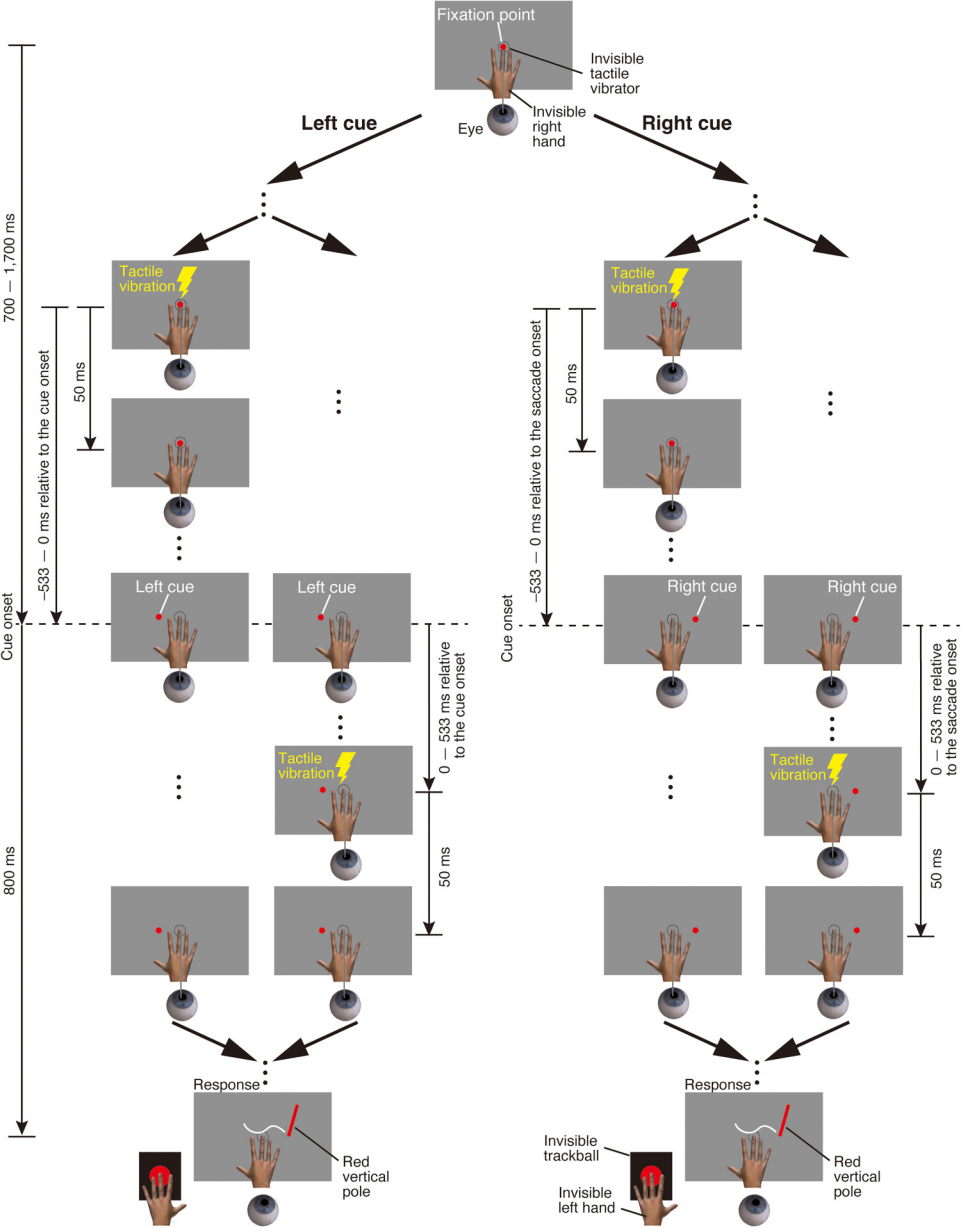
Stimulus sequence in the test condition of Experiment 3. Participants maintained fixation on a central fixation point. After a randomly selected delay between 700 and 1,700 ms, the fixation point disappeared and a cue appeared, either to the left or right of the fixation point. Participants were instructed to maintain fixation while directing their attention to the cue. A tactile stimulus was then presented for 50 ms. Its timing relative to cue onset was randomly selected from 13 time points: ±533, ±444, ±356, ±267, ±178, ±89, and 0 ms. After the cue disappeared, a red vertical pole appeared. Participants localized the perceived position of the tactile stimulus by moving the red vertical pole using a trackball.

The procedure of Experiment 3 was identical to that of Experiment 2 except in the following aspects (Figure 11). Two conditions were used, namely, a control condition and a test condition, which were identical to the fixation condition and eye-movement condition in Experiment 2, respectively. Note that participants did not make a saccade in either the control or test condition. In the test condition, the participant fixated on the fixation point and pressed a button to start a trial. After a randomly selected delay of between 700 and 1,700 ms, the fixation point disappeared, and a red cue (0.4° in diameter, 15.4 cd/m^2^) appeared to the left (−12°) or right (12°). Participants were instructed to maintain fixation and direct their attention to the cue when the fixation point disappeared. Eye position was continuously monitored throughout the trials using the eye tracker. Trials in which their gaze was not maintained within ±2° of the initial fixation position were considered invalid. However, all trials met this criterion. The timing of the tactile stimulus presentation was randomly selected relative to the cue onset time: ±533, ±444, ±356, ±267, ±178, ±89, and 0 ms. Each participant performed six blocks. Within each block, 52 trials (13 timings of the tactile stimulus × 2 cue directions × 2 repetitions) were conducted in a random order. As a result, each timing of the tactile stimulus was presented 12 times per cue direction in total across all blocks (2 repetitions per block × 6 blocks).

### Results and Discussion

**Figure 12.**
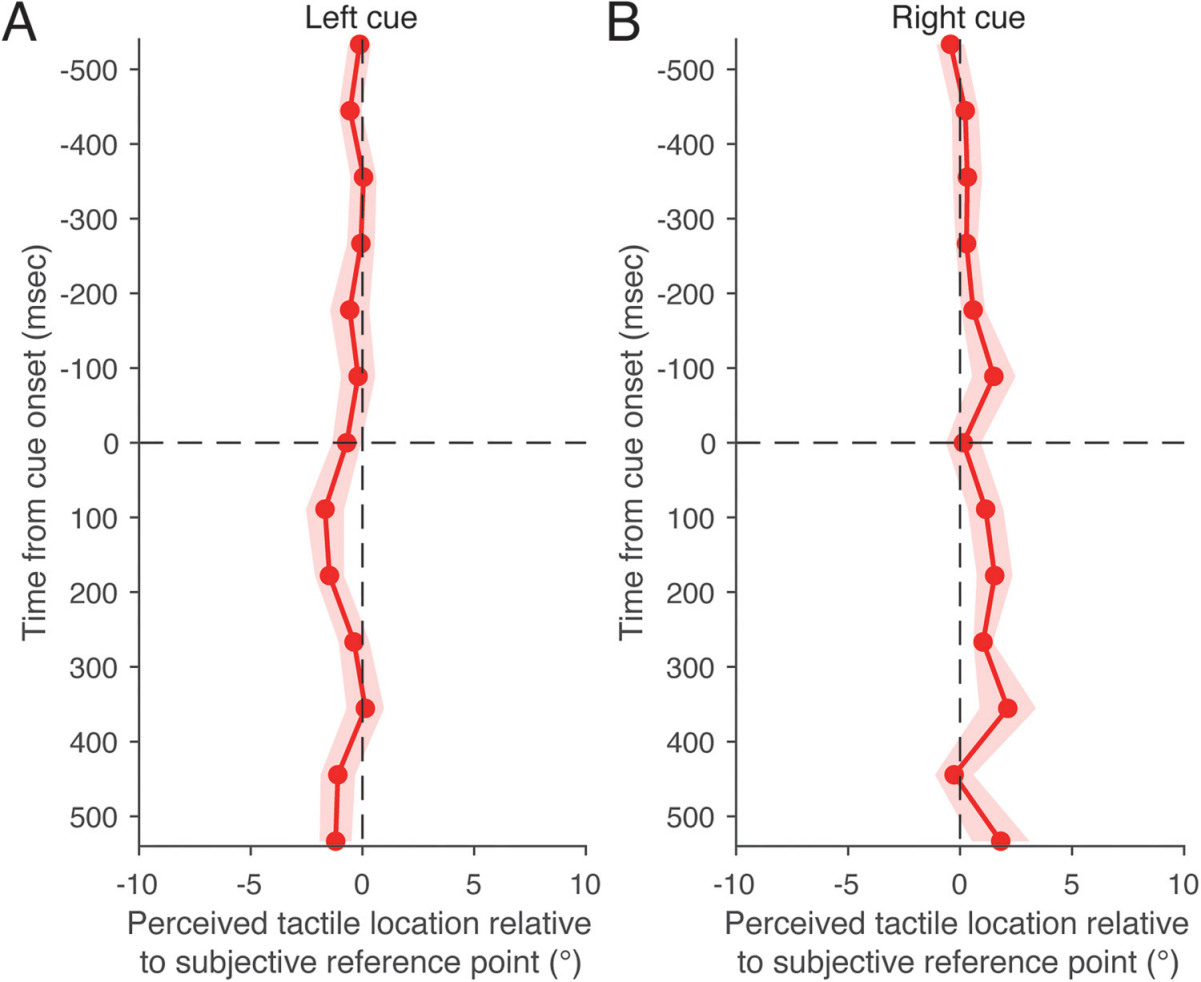
Perceived horizontal location of tactile stimuli as a function of time from cue onset when directing attention to a cue during a fixation in Experiment 3. (A) Left cue. (B) Right cue. Positive values of the perceived tactile location indicate a right side relative to the subjective reference point. Results are the mean ± standard error.

Figure 12 shows the mean perceived horizontal tactile location as a function of time relative to cue onset. This temporal profile was compared with that of the saccade condition, as measured in Experiment 2. There was a significant interaction between condition (saccade vs. attention) and direction (left vs. right), where direction refers to saccade direction in the saccade condition and cue direction in the attention condition (*F*(1, 29) = 8.76, *p* < .01, *f* = 0.55). This interaction was tested as a single term in the statistical analysis. Significant main effects were also found for direction (*F*(1, 29) = 17.12, *p* < .01, *f* = 0.77) and stimulus timing (*F*(12, 348) = 1.93, *p* < .05, *f* = 0.26). A significant interaction was also found between direction and stimulus timing (*F*(12, 348) = 5.34, *p* < .05, *f* = 0.43). A simple main effect analysis revealed that the perceived horizontal tactile location varied significantly depending on saccade direction (*F*(1, 29) = 25.19, *p* < .01, *η^2^* = 0.47), but not on cue direction (*F*(1, 29) = 0.69, *p* = .41, *η^2^* = 0.023). These results indicate that the effect of attention on tactile localization was much weaker than that of saccadic eye movements, consistent with previous findings (Harrar and Harris 2009). Thus, these results suggest that the perceptual shift in tactile localization toward the saccade target is not due to the redirection of attention toward the target.

## General Discussion

This study investigated the effects of a saccadic eye movement on tactile localization. Experiment 1 showed that the perceptual location of a touch shifts in a saccade direction even before the saccade. Experiment 2 demonstrated that this perceptual shift is not due to a bias formed by a repeated saccade in the same direction. Finally, Experiment 3 found that the shift in the perceived location of a touch during a saccade cannot be explained by shifts in attention. Taken together, these results suggest that perceived tactile locations are influenced by saccade planning or preparation, rather than by the execution of the saccadic eye movement itself.

The present results suggest that the time course of tactile localization during a saccade may differ from what has been reported for visual localization. Perceived tactile locations began to shift in the saccade direction about 250 ms before the saccade target was presented. This perceptual shift continued until 500 ms after the saccade onset. In contrast, previous studies have reported that perceived visual locations start to shift approximately 100 ms before the saccade onset, and this shift disappears more than 100 ms after the saccade onset (Dassonville et al. 1995; Honda 1991). Taken together with previous findings, these results may indicate that saccadic eye movements have different effects on tactile and visual localization. Interestingly, Overvliet et al. (2011) reported that saccadic responses to tactile stimuli delivered to the hands were slower when the hands were crossed than when they were uncrossed. This posture-dependent effect on saccades may further support the view that saccades influence tactile and visual localization differently. However, we acknowledge that these comparisons are indirect, as they involve different experimental designs, tasks, and participants.

These differing effects suggest that touch and vision may be coded differently within a visual reference frame. The standard theory of multisensory integration posits that the visual reference frame serves as a single unified reference frame for mapping spatial information from all sensory modalities in the brain (Pouget, Deneve, et al. 2002; Pouget, Ducom, et al. 2002). Indeed, when the eye position is maintained away from straight ahead, a systematic shift occurs in the perceived location of a visual stimulus (Harris and Smith 2008), an auditory stimulus (Lewald and Ehrenstein 1996; Weerts and Thurlow 1971), and a tactile stimulus (Harrar and Harris 2009). These eye-position effects support the view that multisensory spatial information is coded in a visual reference frame. However, previous studies have assumed that spatial information from all sensory modalities must be transformed into a visual reference frame to construct a single unified frame of reference. Although the present findings that saccades affect tactile localization support the view that tactile information is coded in a visual reference frame, this study does not seem to support the view that a visual reference frame is a single unified frame of reference. While our findings are restricted to the tactile domain, the observed differences in localization biases and their respective time courses between touch and vision suggest that these modalities may rely on distinct processing characteristics. However, the present findings cannot determine whether these differences reflect variations within a single common visual reference frame or the use of distributed visual reference frames. Further research is needed to test these possibilities.

The results of this study demonstrated that saccades cause a much greater shift in perceived tactile location than attention. When participants directed their attention to a cue presented in the peripheral visual field while maintaining fixation at the center of the display, the perceived location of a touch was only slightly shifted toward the presented cue. This suggests that attention accounts for a small amount of the shift in perceived tactile location. Indeed, previous studies have shown that perceived tactile locations shift toward an attended region (Flach and Haggard 2006; Kilgard and Merzenich 1995). Regarding the reason for the small shift in perceived tactile location, it is possible that attention was divided between the fixation position and the cue position. However, the present study indicated that the shift in perceived tactile location occurred well before the saccade target was presented while participants fixated on the center of the display. This shift could not be explained by attention, because no target for attention was presented. Thus, these results suggest that there is more to the effect of a saccade on tactile location than attention alone.

How can we explain the shift in perceived tactile location that occurs well before the presentation of the saccade target? One possibility is that this shift results from a bias. In Experiment 1, the saccade direction was fixed in the same direction throughout each block. Therefore, repeated saccades in the same direction may have resulted in a bias in perceived tactile locations in the same direction as the saccade. To eliminate this bias, the saccade direction was randomized for each trial in Experiment 2. Nevertheless, the shift in perceived tactile location still occurred in the same direction as the saccade well before the presentation of the saccade target. Thus, this shift cannot be explained as a bias resulting from repeated saccades in the same direction throughout a single block.

Alternatively, tactile spatial memory may be distorted during saccades. In this study, participants had to memorize the perceived location of a touch. To respond with this memorized tactile location information, they had to wait for the presentation of a response bar stimulus after a saccade. The memorized tactile location may have been affected by saccades. Such spatial memory involves working memory (Burton and Sinclair 2000; Gallace and Spence 2009). Working memory has been reported to assist in the detection and identification of changes in the world that occur during a saccade (Aagten-Murphy and Bays 2019; Bays and Husain 2008). Neurophysiological studies have suggested that perceptual space may be distorted in working memory before saccades (Zirnsak and Moore 2014; Zirnsak et al. 2014). Based on these findings, we speculate that tactile working memory for spatial locations might be distorted well before a saccade. This possibility needs to be investigated in future research.

The present study used a trackball for the tactile localization task. One potential limitation of using this method is that the trackball may require more time to perform the task than a motor response such as a hand-pointing, which could potentially introduce memory-related distortions in the perceived location of the tactile stimulus. To minimize this concern, participants were explicitly instructed to respond as quickly as possible after stimulus presentation. Moreover, the use of a trackball was motivated by our focus on perceptual, rather than motor, localization. Previous studies have shown that hand-pointing tends to indicate the veridical location of a tactile stimulus, even when the perceived location is shifted from the physical location (Dijkerman and de Haan 2007; Kammers et al. 2009; Marcel 2003), which suggests that hand-pointing may not reflect perceptual localization. Therefore, although we recognize the possibility of memory-related effects, we believe that the trackball provided a more appropriate and controlled method for capturing perceptual judgments.

In the present study, a single tactile stimulus was presented to the same finger location while hand posture remained constant across trials. This raises the possibility that participants may have treated the tactile stimulus as a cue and localized the associated body part (i.e., the finger) in external space, rather than remapping the tactile input itself. In this case, the task might primarily assess postural matching during saccades, driven more by proprioceptive information than by tactile spatial remapping. Indeed, previous studies have shown that saccade performance is modulated by hand posture (Overvliet et al. 2011), suggesting a functional link between saccades and hand posture. This link may lead to shifts in perceived hand posture toward the saccade direction. To more directly engage tactile remapping mechanisms during saccades, future studies may need to introduce trial-by-trial variation of the tactile stimulus location in anatomical coordinates.

In conclusion, the findings of this study indicate that the perceived location of a touch shifts toward the direction of a saccade even before the saccade occurs. These results contribute to our understanding of how tactile spatial perception is dynamically modulated in anticipation of oculomotor actions, highlighting the integration between tactile and oculomotor systems in spatial coding.
